# Characterization of an IncFII Plasmid Encoding NDM-1 from *Escherichia coli* ST131

**DOI:** 10.1371/journal.pone.0034752

**Published:** 2012-04-12

**Authors:** Rémy A. Bonnin, Laurent Poirel, Alessandra Carattoli, Patrice Nordmann

**Affiliations:** 1 Service de Bactériologie-Virologie, INSERM U914 «Emerging Resistance to Antibiotics», Hôpital de Bicêtre, Assistance Publique/Hôpitaux de Paris, Faculté de Médecine et Université Paris-Sud, Paris, France; 2 Department of Infectious, Parasitic and Imunno-Mediated Diseases, Istituto Superiore di Sanità, Rome, Italy; Institut National de la Recherche Agronomique, France

## Abstract

**Background:**

The current spread of the gene encoding the metallo-ß-lactamase NDM-1 in *Enterobacteriaceae* is linked to a variety of surrounding genetic structures and plasmid scaffolds.

**Methodology:**

The whole sequence of plasmid pGUE-NDM carrying the *bla*_NDM-1_ gene was determined by high-density pyrosequencing and a genomic comparative analysis with other *bla*_NDM-1_-negative IncFII was performed.

**Principal Findings:**

Plasmid pGUE-NDM replicating in *Escherichia coli* confers resistance to many antibiotic molecules including β-lactams, aminoglycosides, trimethoprim, and sulfonamides. It is 87,022 bp in-size and carries the two β-lactamase genes *bla*_NDM-1_ and *bla*_OXA-1_, together with three aminoglycoside resistance genes *aacA4*, *aadA2*, and *aacC2*. Comparative analysis of the multidrug resistance locus contained a module encompassing the *bla*_NDM-1_ gene that is actually conserved among different structures identified in other enterobacterial isolates. This module was constituted by the *bla*_NDM-1_ gene, a fragment of insertion sequence IS*Aba125* and a bleomycin resistance encoding gene.

**Significance:**

This is the first characterized *bla*_NDM-1_-carrying IncFII-type plasmid. Such association between the *bla*_NDM-1_ gene and an IncFII-type plasmid backbone is extremely worrisome considering that this plasmid type is known to spread efficiently, as examplified with the worldwide dissemination of *bla*_CTX-M-15_-borne IncFII plasmids.

## Introduction

Metallo-ß-lactamase (MBL) NDM-1 (New Dehli Metallo-ß-lactamase) corresponds to one of the latest and most important resistance trait identified in Gram-negative rods [1]. The *bla*_NDM-1_ gene has been first identified in the UK, India, and Pakistan, either in various enterobacterial isolates, but also in *Acinetobacter* sp., *Pseudomonas* sp., or *Vibrio* sp. [2]–[6]. Occurrence of NDM-1 producers in hospitalized patients in the UK was related in many cases with previous hospitalizations in the Indian subcontinent [2]. Additionally, there have been numerous reports of NDM-1-producing *Enterobacteriaceae* worldwide (infections or colonizations), most often recovered from patients who presented a link with the Indian subcontinent, and in some cases with the Balkans and Middle-East region [6]–[11].

Five *bla*_NDM-1_-bearing plasmids have been fully sequenced, being pHK-NDM (Genbank n°HQ451074), p271A (JF785549), pNDM-1_Dok01 (AP012208), pNDM10505 (JF503991) and pNDM-KN (JN157804), respectively corresponding to IncL/M-, IncN2-, and IncA/C-type plasmid scaffolds [12]–[16]. pHK-NDM is an IncL/M 88,803-bp in-size plasmid harboring the *bla*_NDM-1_ gene together with the 16S rRNA methylase *armA* gene, thus conferring high level of resistance to β-lactams and aminoglycosides, and is highly related to plasmids carrying the *bla*_CTX-M-3_ gene [12]. Plasmid p271A is 35,947-bp in size and harbors the *bla*_NDM-1_ gene as a single resistance gene within an IncN2-type plasmid scaffold on which a new replicase gene was identified [13]. Plasmids pNDM-1_Dok01, pNDM10505 and pNDM-KN belong to the IncA/C broad-host range plasmid family. In addition to the *bla*_NDM-1_ gene, those plasmids carry additional resistance genes including a *bla*_CMY-2_-like gene, together with an *rmtC* or *armA* 16S RNA methylase gene. Their scaffolds are very similar to those of other IncA/C but *bla*_NDM-1_-negative plasmids, known to be responsible for the spread of *bla*_CMY-2_-like genes in *Enterobacteriaceae* in the USA, Canada, and Europe [14], [15].

Overall, the *bla*_NDM-1_ gene has been more frequently reported onto broad host-range IncA/C-type plasmids, either in clinical or in environmental isolates recovered from the New Delhi area [6]. In addition, some other plasmid scaffolds have been associated with the *bla*_NDM-1_ gene including IncF, IncL/M, together with untypeable plasmids [6], [8]. In particular, IncF-type plasmids were involved in *bla*_NDM-1_ acquisition in isolates recovered in France, India, and Switzerland [8].

IncFII-type plasmids are narrow-host range plasmids that are frequently identified among *E. coli* strains [16]. Those plasmids are known to be involved in the worldwide dissemination of the *bla*_CTX-M-15_ gene in the epidemic ST131 *E. coli* clone [17]. They are characterized by several toxin-antitoxin addiction systems confering stability during bacterial cell division. The IncFII-type pC15-1a (GenBank n° NC_005327) and pEK516 (EU935738) plasmids carrying the *bla*_CTX-M-15_ gene have been fully sequenced [17], [18]. In particular, plasmid pEK516 obtained from the UK epidemic CTX-M-15-producing strain D represents the prototypic IncFII-plasmid from the MLST-defined ST131 *E. coli* lineage [18]. Unlike plasmids belonging to other incompatibilty groups, the backbones of IncF plasmids exhibit a significant heterogeneity in term of size and number of replicons [19].

The aim of this study was to characterize in detail an IncFII-type plasmid harboring the *bla*_NDM-1_ gene, in order to possibly identify genetic features explaining the successful spread of that gene. That plasmid had been recovered from an *E. coli* isolate belonging to ST131 [20] that corresponds to the main genetic background involved in the worldwide distribution of CTX-M-15 producers [21].

## Results

### General features

Our study was initiated by the isolation of a multidrug-resistant *E. coli* strain GUE that had been community-acquired in India [20]. *E. coli* isolate GUE was resistant to most β-lactams (remaining susceptible to aztreonam) and showed reduced susceptibility to carbapenems, minimum inhibitory concentrations (MIC) of imipenem, ertapenem, and meropenem being at 3, 3 and 2 µg/ml, respectively (Table 1). It was also resistant to gentamicin, kanamycin, tobramycin, sulfonamides, tetracycline, and fluoroquinolones, but remained susceptible to amikacin, chloramphenicol, rifampicin, and colistin.

**Table 1:** MICs of ß-lactams for *E. coli* clinical isolate Gue, *E. coli* J53 pGUE-NDM and *E. coli* J53 reference strains.

β-lactams	MIC (µg/ml)		
	*E. coli* isolate GUE	*E. coli* J53 pGUE-NDM	*E. coli* J53
Amoxicillin	>256	>256	4
Amoxicillin + CLA^a^	256	>256	4
Ticarcillin	>256	>256	2
Ticarcillin + CLA^a^	256	256	2
Cephalothin	>256	>256	4
Cefotaxime	128	128	0.06
Ceftazidime	>256	>256	0.06
Cefepime	32	32	0.03
Aztreonam	0.125	0.06	0.06
Meropenem	2	0.75	0.03
Ertapenem	3	0.5	0.03
Doripenem	1.5	0.5	0.03
Imipenem	3	1.5	0.12

a CLA; clavulanic acid (4 µg/ml) TZB.

### Plasmid features

Conjugation assays allowed to transfer the *bla*_NDM-1_ gene and identified a ca. 87-kb plasmid named pGUE-NDM. The *E. coli* transconjugant showed resistance to penicillin/inhibitor combinations and broad-spectrum cephalosporins, and reduced susceptibilty to carbapenems (Table 1). It was also resistant to kanamycin, gentamicin, tobramycin, trimethoprim, and sulfonamides. Plasmid pGUE-NDM was assigned to the IncFII incompatibility group using the PCR-Based Replicon Typing method. Conjugation frequencies were observed at high rates (2×10^−4^ transconjugants per donor cell).

### Sequence analysis of plasmid pGUE-NDM

**Figure 1:**
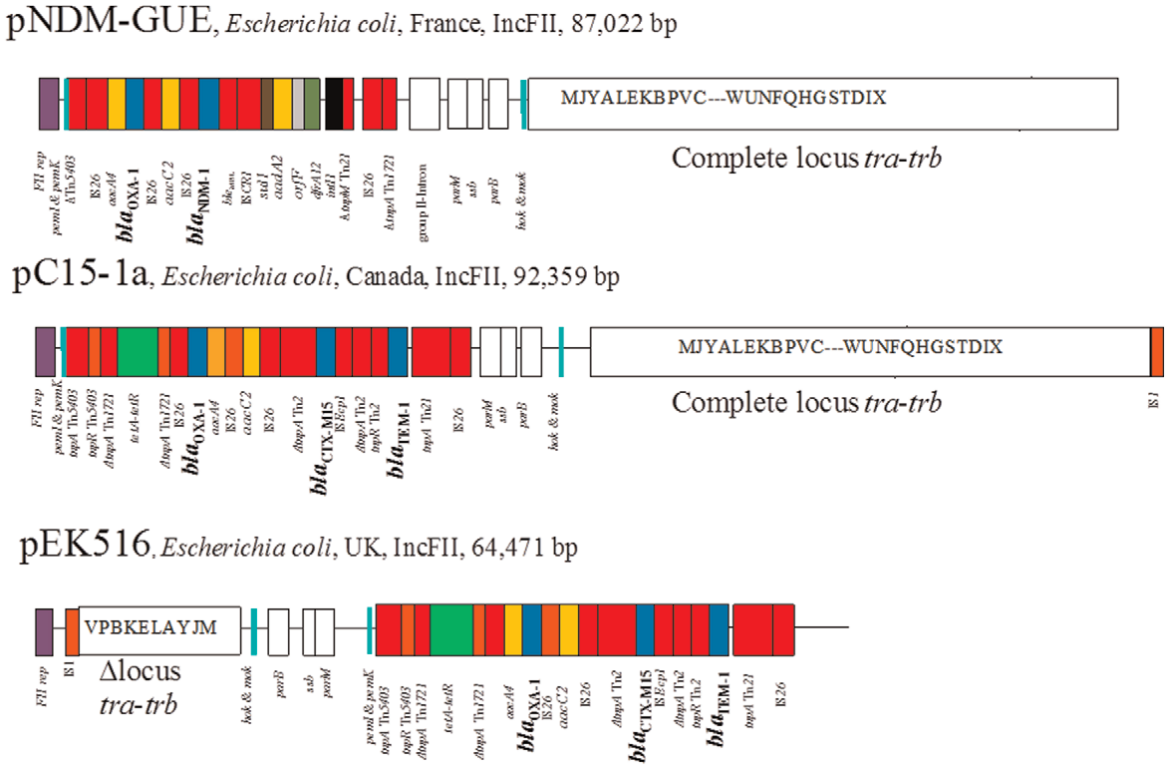
Major structural features of pGUE-NDM, encoding NDM-1 MBL, in comparison with incFII plasmids pC15-1a (genbank n°AY458016) and pEK516 (Genbank n°EU935738) CTX-M-15 encoding plasmids. Resistance genes are indicated by colored boxes as follows: green, tetracycline resistance (*tetA*, *tetR*); azure, β-lactams resistance genes (*bla*_NDM-1_, *bla*_OXA-1_, *bla*_CTX-M-15_, *bla*_TEM-1_); orange, aminoglycosides resistance genes (*aacA4*, *aacC2*, *aadA2*); dark green, trimethoprime resistance gene (*dfrA12*). Transposon-related genes and insertion sequences are indicated in red boxes. Class 1 integrase gene is indicated by a black box. Partitioning-associated genes (*parB*, *parM*, *ssb*) are indicated by black-squared white boxes. The locus *tra-trb* is indicated by a squared white box with capital letters indicating the respective genes. The toxin-antitoxin genes (*pemI/pemK*, *hok/mok*)are indicated by light blue vertical lines. Replicons are indicated by purple boxes.

Whole plasmid sequencing identified plasmid pGUE-NDM to be 87,022 bp in-size with a GC content of 53% and 47 open reading frames (ORF) (Figure 1 and Table S1). BLAST analysis of the complete nucleotide sequence was performed in comparison with the reference IncFII plasmid pC15-1a (Genbank n°AY458016) and with plasmid pEK516 UK (Genbank n° EU935738). Comparative DNA sequence analysis confirmed that pGUE-NDM possessed an IncFII-type backbone and exhibited a significant synteny within the two scaffolds, with the exception of regions containing accessory genes (Table S1).

The backbone of pGUE-NDM included a complete array of genes involved in replication, conjugation and partition. The replication operon was composed by four ORFs exhibiting high identity with IncFII-specific replicase genes. The toxin/antitoxin addiction systems system *pemI*/*pemK* and *hok/mok* were identified as previously described for other FII-plasmids (Figure 1), together with the *parB*, *parM* genes encoding proteins involved in plasmid stability (Figure 1 and Table S1). A complete transfer operon (locus *tra-trb*) was identified being involved in the plasmid dissemination.

**Figure 2:**
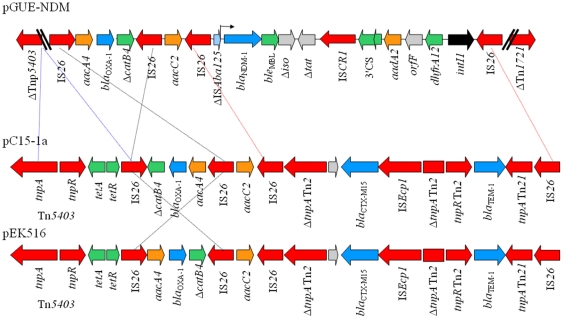
Schematic representation of multi-drug resistance regions of pGUE-NDM, pC15-1a and pEK516 incFII plasmids. The genes and their corresponding transcriptional orientations are indicated by horizontal arrows. Function of each genes are indicated by colored arrows as follows: green, tetracycline resistance; azure, β-lactams resistance genes; orange, aminoglycosides resistance genes; dark green, trimethoprim resistance gene. Transposon-related genes and insertion sequences are indicated in red arrows. Class 1 integrase gene is indicated by black arrow. Deletion is indicated by blue dashed lines. Homologous recombination event leading to inversion is indicated by black dashed lines. Homologous recombination event leading to allelic exchange of *bla*_CTX-M-15_ locus by *bla*_NDM-1_ locus is indicated by red dashed lines. The accession numbers were: pC15-1a (genbank n°AY458016) and pEK516 (genbank n°EU935738).

**Figure 3:**
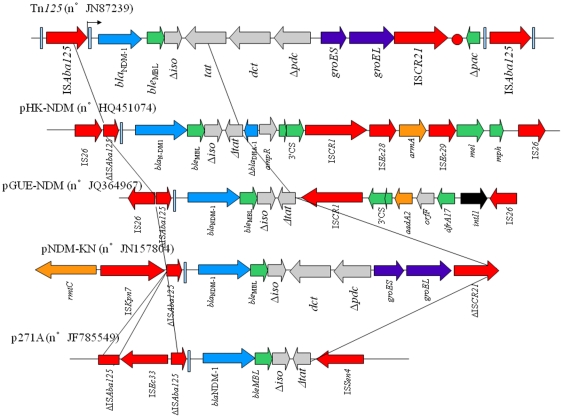
Schematic representation of the DNA sequences surrounding the *bla*_NDM-1_ genes in *Acinetobacter baumannii* strain ML, *E. coli* HK-NDM NDM-1 encoding plasmid pHK-NDM, *E. coli* 271 p271a NDM-1 harboring plasmid, *K. pneumoniae* pNDM-KN harboring plasmid, and *E. coli* GUE NDM-1 harboring pGUE-NDM plasmid. Gene names are abbreviated according to their corresponding proteins: *Δiso* for phosphoribosylanthranilate isomerase; *tat* for twin-arginine translocation pathway signal sequence protein; *dvt* for divalent cation tolerance protein; *Δpac* for truncated phospholipid acetyltransferase. The oriIS of ISCR21 is indicated by a circle. Function of each genes are indicated by colored arrows as follows: green, non-aminoglycosides non-β-lactams resistance genes; azure, β-lactams resistance genes; orange, aminoglycosides resistance genes. Transposon-related genes and insertion sequences are indicated in red arrows. Class 1 integrase gene is indicated by black arrow. GroES, GroEL are indicated in dark blue. ORF of unknown function are indicated by grey arrows. Conserved *bla*_NDM-1_ locus was indicated by vertical black lines. The Genbank accession numbers were: pHK-NDM n°HQ451074, p271A n°JF785549, pNDM-KN pending, pGUE-NDM pending and Tn*125* pending.

The *bla*_NDM-1_ gene was localized in a multidrug resistance (MDR) region of 20,181 bp. This region was bracketed by two copies of insertion sequence IS*26* in opposite orientations creating an IS*26*-made compound transposon that was not bracketed by a target site duplication. Immediately upstream of the *bla*_NDM-1_ gene, a remnant of IS*Aba125* insertion sequence was identified (Figure 2). This truncated IS*Aba125* contained the −35 promoter sequence leading to the *bla*_NDM-1_ gene expression [13]. Upstream of the ΔIS*Aba125*, two IS*26* were identified bracketing the *aacC2* aminoglycoside resistance gene. Then, three gene cassettes being part of a remnant of a class 1 integron, namely *aacC4*, *bla*_OXA-1_ and a truncated *catB4* genes were identified, that latter gene being truncated by another IS*26* element (Figure 2). Downstream of the *bla*_NDM-1_ gene, the *ble*_MBL_ gene encoding resistance to bleomycin was identified followed by a truncated phosphorybosilanthranilate isomerase gene (Δ*iso*), and then by a truncated twin-arginine translocation pathway signal protein gene (Δ*tat*), as previously found on other plasmid scaffolds (Figure 3). Then, the IS*CR1* insertion sequence was identified [22], followed by a class 1 integron structure containing three gene cassettes, namely *aadA2* encoding resistance to streptomycin and spectinomycin, *orfF* of unknown function, and *dfrA12* encoding resistance to trimethoprim, and then the class 1 integrase gene. Finally, an additional copy of IS*26* truncating the Tn*1721* transposase gene was identified (Figure 2). Analysis of surrounding sequences of each IS*26* elements revealed that none of them was bracketed by direct repeat (DR) sequences. Similarly, no DR was identified that would suggest that some of these elements may form an IS*26*-made transposons. This result reinforced the hypothesis of successive homologous recombination events at the origin of the global structure of this MDR region.

### Evolution of the *bla*_NDM-1_ genetic context

In order to get further insights into the *bla*_NDM-1_ gene acquisition, a comparison of the genetic structures previously identified with that identified on plasmid pGUE-NDM was performed (Figure 3). Interestingly, a common module that we termed NDM module was systematically identified. This module was composed of the IS*Aba125* fragment containing the −35 promoter region, the *bla*_NDM-1_ gene, the bleomycin resistance gene *ble*_MBL_, and a truncated phosphoribosylanthranilate isomerase. Recently, composite transposon Tn*125* has been identified in several *A. baumannii* isolates [23], [24]. This 10,099-bp in-size transposon of was made of two copies of IS*Aba125* bracketing a 7,925-bp fragment and was inserted into the chromosome of different strains (Figure 3). Transposon Tn*125* also contained the NDM module, together with the *groES* and *groEL* genes and the IS*CR21* insertion sequence (Figure 3). It seems that the NDM module has integrated the pGUE-NDM backbone by a recombination event mediated by IS*26* elements. Overall, a series of IS*26*-mediated recombination events has likely been at the origin of the formation of the large resistance gene array identified on plasmid pGUE-NDM.

## Discussion

The *bla*_NDM-1_ gene is now identified worldwide and it has been speculated that particular genetic features could be at the origin of that wide diffusion. After the characterization of IncA/C, IncN2, and IncL/M plasmids bearing the *bla*_NDM-1_ gene, our study characterized an IncFII plasmid which backbone is known to be a major vehicle for dissemination of the *bla*_CTX-M-15_ gene [17]. It might be hypothetized that the endemicity of the *bla*_CTX-M-15_-positive and IncFII plasmid in the Indian subcontinent has created a favorable context for hosting and therefore disseminating the *bla*_NDM-1_ gene. That finding is particularly threatening when considering the explosive diffusion of the *bla*_CTX-M-15_ gene that has been witnessed worldwide during the last decade.

Analysis of the genetic features of plasmid pGUE-NDM did not identify any particular element that would positively or negatively interfere into its spreading potency such as addiction systems, partitioning systems or virulence factors in comparison to others IncFII-type plasmids. Indeed, the backbone part of the plasmid (being replication system, maintenance systems and transfer systems) corresponded to one previously identified. However, it appeared that acquisition of the *bla*_NDM-1_-containing module resulted from a series of IS*26-*related recombination events. Our study further underlines that the current dissemination of the *bla*_NDM-1_ gene is associated to a variety of genetic backgrounds.

## Materials and Methods

### Antimicrobial agents and MIC determinations

Susceptibility testing was performed by disk diffusion assay (Sanofi-Diagnostic Pasteur, Marnes-la-Coquette, France) and interpreted aaccording to CLSI [25]. The MICs for carbapenems were determined by Etest (AB Biodisk, Solna, Sweden) on Mueller-Hinton agar plates at 37°C. The production of MBLs was evaluated using Etest, combining imipenem and EDTA as recommended by the manufacturer (AB bioMérieux).

### Plasmid identification and preparation

Plasmid pGUE-NDM was assigned to the IncFII incompatibility group using the PCR-Based Replicon Typing (PBRT)method [26]. Conjugation experiments were performed as previously described using azide-resistant *E. coli* strain J53 [27]. Transconjugants were selected on Trypticase-Soy media containing 100 µg/ml of ticarcillin and 100 µg/ml of sodium azide.

### High-density pyrosequencing and sequence assembly

Plasmid DNA was isolated from the *E. coli* tranconjugant using Qiagen Maxiprep kit (Qiagen, Courtaboeuf, France). The complete sequencing work flow of the Illumina Genome Analyzer IIx system (Illumina Inc., San Diego, CA) was performed by the DNAVision company (Gosselles, Belgium) and is described at www.dnavision.com.

### Genome assembly and annotation

Reads from each sample were trimmed to remove poor quality sequence using the following procedure: keeping only reads for which the quality of all the fifty first bases is greater or equal to ten in both reads; for each read trimming the tail of the sequence from the first position for which the quality is smaller than ten; trimming poly-A sequence artifacts; and trimming adapter sequences. Then, the assemblies were carried out using Velvet2 assembler [28] in order to produce contigs from Illumina GAIIx reads. A total of ca. 12,000 high-quality reads were derived from the pGUE-NDM library, of which 482 contig were obtained using Velvet2 assembler including 14 contigs *de novo*. Contigs were considered as DNA contamination if showing at least 90% similarity with *E. coli* K12 substrain MG1655 genomic DNA sequences (NCBI accession number NC_000913.2) using the BLASTn algorithm. Among the 482 contigs, 430 were considered as DNA contaminations. A single contiguous sequence was obtained from the 52 contigs obtained using PCR-based gap closure et PCR combinations to close gaps and verify the position of each contig.

### Genome comparison

The BLASTp algorithm was used to search for protein similarities by using as a reference the *E. coli* K12 chromosome sequence. The criterion used to evaluate the deduced amino acid sequence homology was >50% similarity at the amino acid level and >50% coverage of protein length.

### Nucleotide sequence accession numbers

The pGUE-NDM plasmid sequence was submitted to the GenBank database and can be found under accession number JQ364967.

### Transparency declaration

None to declare.

## Supporting Information

Table S1 **Orfs identified in pGUE-NDM.**
(DOC)
