# Supplementary material for: Characterization of an IncFII Plasmid Encoding NDM-1 from Escherichia coli ST131
Source: PLoS One. 2012 Apr 12;7(4):e34752. doi: 10.1371/journal.pone.0034752 (PMC3325265; doi:10.1371/journal.pone.0034752)
Supplement: Table S1 — Orfs identified in pGUE-NDM. (DOC) [file pone.0034752.s001.doc]

Table S1. Orfs identified in pGUE-NDM.

| Gene name | Position  (without stop codon) | Length of the corresponding protein  (amino-acids) | Function encoded | Amino-acid identity |
| --- | --- | --- | --- | --- |
| *repA4* | Compl. 4-387 | 128 | Regulation of replication | 98% IncFII RepA4 pC15-1a |
| *orf1* | Compl. 520-687 | 56 | unknown | 100% (EFK71827.1)  *E. coli* MS 78-1 |
| *repA1* | Compl. 753-1619 | 289 | Replication | 100% IncFII RepA1  *E. coli* strain L8 |
| *repA3* (*copA*) | Compl. 1623-1814 | 64 | Regulation of replication | 100% IncFII RepA3  *E. coli* plasmid pL8 |
| *repA2* | Compl. 1915-2166 | 84 | Regulation of replication | 100% IncFII RepA2  plasmid R100 |
| *yihA* | Compl. 2409-2996 | 196 | Unknown | 99% plasmid R100 (23-196) |
| *yigB* | Compl. 3292-3750 | 153 | Putive nuclease domain protein | 99% *E. coli* ETEC h10407 |
| *orf2* | Compl. 3951-4202 | 84 | Unknown | 90% *E. coli* ETEC h10407 |
| *orf3* | Compl. 4863-5537 | 225 | Unknown | 100% ECO26_p2-80  *E. coli* ECO26 |
| *finO* | Compl. 6170-6727 | 186 | Fertility inhibition protein | 99% FinO  *E. coli* STEC_MHI813 |
| *traX* | Compl. 6785-7732 | 316 | F-pilin acetylation protein | 91% TraX  *E. coli* M605 |
| *traI* | Compl. 7551-12818 | 1756 | Conjugal transfer nickase/helicase | 99% TraI  *E. coli* E22 |
| *traD* | Compl. 12821-15043 | 741 | Conjugal transfer coupling protein | 99% TraD  *E. coli* EC4100B |
| *traT* | Compl. 15299-16051 | 251 | Surface exclusion protein | 99% TraT *E. coli* |
| *traS* | Compl. 16044-16565 | 174 | Surface exclusion protein | 99% TraS *E. coli* |
| *traG* | Compl. 16550-19372 | 941 | Mating contact stabilization protein | 99% TraG  *E. coli* TA271 |
| *traH* | Compl. 19372-20745 | 458 | conjugative transfer system pilus assembly protein | 100% TraH  *E. coli* SMS-3-5 |
| *trbJ* | Compl. 20745-21185 | 147 | Conjugal transfer protein | 97% TrbJ *E. coli* pEC-B24 |
| *trbB* | Compl. 21037-21579 | 181 | pilin assembly thiol-disulfide isomerase | 99% TrbB *E. coli* TW10509 |
| *traQ* | Compl. 21569-21850 | 94 | conjugal transfer pilus chaperone | 99% TraQ *E. coli* UMN026 |
| *trbA* | Compl. 21972-22316 | 115 | conjugative transfer system protein | 99% TrbA *E. coli* TA271 |
| *traF* | Compl. 22335-23075 | 247 | pilus assembly protein | 99% TraF *E. coli* pR100 |
| *traN* | Compl. 23355-25160 | 602 | mating-pair stabilization protein | 100% TraN *E. coli* SMS3-5 |
| *trbC* | Compl. 25160-25798 | 213 | pilus assembly protein | 100% TrbC *E. coli* TA271 |
| *traU* | Compl. 25807-26796 | 330 | pilus assembly protein | 100% TraU *E. coli* TA271 |
| *traW* | Compl. 26796-27539 | 248 | pilus assembly protein | 99% TraW *E. coli* APEC01 |
| *trbI* | Compl. 27425-27808 | 128 | Conjugal transfer protein | 98% TrbI *E. coli* ECO26 p2-55 |
| *traC* | Compl. 27808-30435 | 876 | ATP-binding protein | 99% *E. coli* pVM01-p105 |
| *yfiC* | Compl. 30564-30923 | 120 | Hypothetical protein | 95% YfiC *E. coli* pC15-1a |
| *yfhA* | Compl. 31248-31721 | 158 | Hypothetical protein | 97% YfhA *E. coli* |
| *traR* | Compl. 31717-31935 | 73 | Conjugal transfer regulatory protein | 100% TraR *E. coli* ETEC H10407 |
| *traV* | Compl. 32073-32585 | 171 | Pilus assembly protein | 99% TraV *E. coli* UMNK88 |
| *trbG* | Compl. 32585-32833 | 83 | Conjugal transfer protein | 100% TrbG plasmid F |
| *trbD* | Compl. 32829-33236 | 136 | Conjugal transfer protein | 92% TrbD *E. coli* |
| *traP* | Compl. 33136-33723 | 196 | Conjugal transfer protein | 100% TraP *E. coli* UTI89 |
| *traB* | Compl. 33716-35140 | 475 | pilus assembly protein | 100% TraB *E. coli* SMS3-5 |
| *traK* | Compl. 35143-35868 | 242 | conjugative transfer system secretin | 100% *E. coli* 1520 |
| *traE* | Compl. 35858-36268 | 137 | Pilus assembly protein | 100% TraE *E. coli* MS 110-3 |
| *traL* | Compl. 36446-36754 | 103 | Pilus assembly protein | 100% TraL *E. coli* TA271 |
| *traA* | Compl. 36772-37128 | 119 | Conjugal transfer pilin subunit | 100% TraA *E. coli* SMS-3-5 |
| *traY* | Compl. 37165-37389 | 75 | Conjugal transfer protein | 99% TraY *E. coli* SMS-3-5 |
| *traJ* | Compl. 37486-38229 | 248 | Functional relaxosome complex initiator | 98% TraJ *E. coli* UTI89 |
| *traM* | Compl. 38363-38743 | 127 | Signal transduction protein | 100% TraM *E. coli* SMS-3-5 |
| *X-polypeptide* | 39020-39645 | 208 | Transglycosylase SLT domain protein | 99% X-polypeptide *E. coli* SMS-3-5 |
| *yubP* | Compl. 39966-40784 | 273 | Hypothetical protein | 100% YubB *E. coli* B88 |
| *yubO* | 40851-41231 | 127 | Hypothetical protein | 99% YubO *E. coli* pECB24_p27 |
| *hok* | Compl. 42114-42356 | 81 | Post-segregational killing protein | 99% Hok *E. coli* pECB24 |
| *mok* | 42313-42534 | 74 | Regulator de HOK | 100% Mok *E. coli* pECB24 |
| *psiA* | Compl. 42552-43268 | 239 | Plasmid SOS inhibition protein A | 100% PsiA *E. coli* pECB24 |
| *psiB* | Compl. 43268-43720 | 151 | Plasmid SOS inhibition protein B | 95% PsiB *E. coli* pECB24 |
| *parB-like* | Compl. 43757-45712 | 652 | ParB-like partition protein | 99% Hypothetical protein *E. coli* TA271 |
| *ssb* | Compl. 46077-46718 | 214 | Single-stranded DNA binding protein | 96% Ssb *E. coli* pECB24 |
| *ycdA* | Compl. 47506-48117 | 204 | Hypothetical protein | 99% YcdA *E. coli* pECB24 |
| *ydbA* | Compl. 48117-49475 | 465 | Hypothetical methyltransferase domain protein | 100% Hypothetical protein *E. coli* MS78-1 |
| *ydaB* | Compl. 49530-49757 | 76 | Hypothetical protein | 100% YdaB *E. coli* pR100 |
| *ydaA* | Compl. 50188-50568 | 127 | Hypothetical protein | 99% YdaA *E. coli* pEC_L8 |
| *IntronII* | 51154-52818 | 555 | group II intron-associated polymerase | 100% IntronII *E. coli* pIP1206 |
| *orf4* | Compl. 52939-53271 | 111 | Hypothetical protein | 100% p36 *E. coli* pEK499 |
| *orf5* | Compl. 53326-53775 | 150 | Hypothetical protein | 97% Hypothetical protein *E. coli* MS146-& |
| *klcA* | Compl. 53795-54220 | 142 | Antirestriction protein | 99% KlcA *E.coli* pEC_L46 |
| *ycfA* | Compl. 54580-55347 | 256 | Hypothetical protein | 99% Hypothetical protein *E. coli* E110019 |
| *ycgB* | Compl. 55395-55826 | 144 | Hypothetical protein | 99% Hypothetical protein *E. coli* 3030-1 |
| *yfbB* | Compl. 56064-56744 | 227 | Putative DNA methylase protein | 99% DNA methylase protein |
| *orf4* | Compl. 56824-57246 | 141 | Hypothetical protein | 86% hypothetical protein *E. coli* UMNF18 |
| *parM* | 57261-58220 | 320 | Plasmid segregation protein | 100% ParM *E. coli* TX1999 |
| *stbB* | 58226-58573 | 116 | Stable plasmid inheritance protein B | 98% StbB *E. coli* pR100 |
| *orf5* | 58707-59144 | 146 | Hypothetical protein | 89% Hypothetical Protein *E. coli* ECSE_P2-0101 |
| *orf6* | Compl. 59375-60373 | 333 | Hypothetical protein | 100% Hypothetical Protein *E. coli* ECOK1357_2568 |
| *orf7* | 60676-63348 | 891 | Hypothetical protein | 99% Hypothetical Protein *E. coli* ECOK1357_2569 |
| *orf8* | Compl. 63865-64746 | 294 | Hypothetical protein | 100% Hypothetical Protein *E. coli* ECOK1357_2570 |
| *ΔtnpA* Tn*1721* | Compl. 65176-66329 | 384 | Truncated transposase | 100% TnpA Tn*1721* |
| *tnpA* IS*26* | 66383-67096 | 238 | transposase | 100% TnpA IS*26* |
| *tnpM* Tn*21* | Compl. 67521-68102 | 194 | Transposition modulator protein | 100% TnpM Tn*21* |
| *inti1* | Compl. 68074-69084 | 337 | Class 1 integrase | 100% Class 1 integrase IntI1 |
| *dfrA12* | 69229-69723 | 165 | Dihydrofolate reductase | 100% DhfrA12 |
| *orfF* | 69838-70125 | 96 | Hypothetical protein | 100% OrfF |
| *aadA2* | 70134-70922 | 263 | Aminoglycosides adenyltransferase | 100% AadA2 |
| *qacEΔ1* | 71089-71433 | 115 | Multidrug efflux protein | 100% QacEΔ1 |
| *sul1* | 71343-72266 | 308 | Sulfonamides resistance protein | 100% Sul1 |
| *tnpA* IS*CR1* | 72674-74212 | 513 | transposase | 100% TnpA IS*CR1* |
| *Δtat* | 74993-75556 | 188 | truncated hypothetical twin-arginine translocation pathway signal sequence protein | 100% hypothetical protein *E. coli* p271A |
| *Δiso* | Compl. 75669-76208 | 180 | Putative phosphoribosylanthranilate isomerase | 100% hypothetical protein *E. coli* p271A |
| *bleMBL* | Compl. 76216-76578 | 121 | Bleomycine resistance protein | 100% BleMBL *E. coli* p271A |
| *bla*_NDM-1_ | Compl. 76585-77394 | 270 | Metallo-β-lactamase | 100% NDM-1 |
| *ΔtnpA* IS*Aba125* | Compl. 77498-77773 | 92 | Truncated transposase | 100% TnpA IS*Aba125* |
| *tnpA* IS*26* | 77941-78654 | 238 | transposase | 100% TnpA IS*26* |
| *orf9* | Compl. 78994-79533 | 180 | Hypothetical protein | 100% *E. coli* pC15-1a_013 |
| *aacC3* | Compl. 79549-80406 | 286 | Aminoglycosides acetyltransferase | 100% AAC(3')II |
| *tnpA* IS*26* | 80501-81214 | 238 | transposase | 100% TnpA IS*26* |
| *ΔcatB4* | Compl. 81271-81711 | 147 | Truncated chloramphenicol acetyltransferase | 100% CatB4 |
| *bla*_OXA-1_ | Compl. 81852-82679 | 276 | Narrow spectrum β-lactamase | 100% OXA-1 |
| *aacA4* | Compl. 82813-83409 | 199 | Aminoglycosides acetyltransferase | 100% AAC(6')-Ib |
| *tnpA* IS*26* | Compl. 83513-84226 | 238 | transposase | 100% TnpA IS*26* |
| *ΔtnpA* Tn*5403* | 84279-85355 | 359 | Truncated transposase | 100% TnpA Tn*5403* |
| *pemK* | Compl. 85498-85905 | 136 | Programmed cell death toxin | 100% PemK *E. coli* UMN026 |
| *pemI* | Compl. 85832-86086 | 85 | Programmed cell death anti-toxin | 100% PemK *E. coli* UMN026 |
| *tir* | 86203-86832 | 210 | Transfer inhibition protein | 100% Tir *E. coli* pR100 |
